# Psychoactive substance use among psychiatric in-patients presenting to the Emergency Centre of a district hospital in Cape Town, South Africa. A retrospective descriptive study

**DOI:** 10.1016/j.afjem.2025.02.006

**Published:** 2025-03-21

**Authors:** Nardus Droomer, Paul Xafis, Philip Cloete

**Affiliations:** Division of Emergency Medicine, Department of Family, Community and Emergency Care, Faculty of Health Sciences, University of Cape Town, Cape Town, South Africa

**Keywords:** Emergency Centre, South Africa, Mental illness, Substance use, Diagnosis

## Abstract

**Background:**

Mental illness and substance use are major global challenges, with their impact on Emergency Centres becoming evident, especially in South Africa. Patients facing these issues require significant resources from both hospital and community services. However, there is a lack of local data regarding the prevalence of concurrent mental health and substance use disorders. This study aims to evaluate the extent of psychoactive substance use within the psychiatric population at a District Hospital in Cape Town, South Africa.

**Methods:**

This study is a single-centre, retrospective descriptive analysis. It includes all patients referred to the inpatient psychiatric service over six months, recorded in an electronic database. Data were statistically analysed, considering the following variables: urine drug test results to identify specific substance (s) used, sex, age, diagnosis, and repeat visits.

**Results:**

A total of 597 patient visits were analysed. Fifty-nine percent tested positive for at least one substance. The patients’ average age was 34 years. A greater percentage of visits were for males (58 %), with males exhibiting a higher rate of positive test results (64 %) than females (51 %). Among the 146 repeat visits, a significant association was found between the number of visits and positive test results, with 73 % of patients with ≥2 repeat visits testing positive for substances (*p* < 0.001). Cannabis (60 %), methamphetamines (47 %), benzodiazepines (26 %), opioids (7 %), and cocaine (1 %) were the substances most frequently reported.

**Conclusions:**

Emergency Centres in South Africa are impacted by individuals seeking mental health care, and substance use significantly exacerbates these challenges. Substance use creates serious physical, mental, and social implications for patients. As emergency care practitioners and members of the broader healthcare system, we play vital roles in addressing these issues. This study provides valuable insights into the complexities of the situation and suggests potential approaches for intervention.

**African relevance**

• This article discusses the increasing burden of mental health and substance use disorders in Low and Middle-Income Countries (LMICs), where >80 % of individuals with mental health disorders live.
• In Africa, these issues are exacerbated by under-resourced healthcare systems, limited access to mental health services, and social stigma surrounding mental illness and substance abuse.
• The impact of co-occurring mental illness and substance abuse on emergency care and psychiatric units is particularly evident in African nations like South Africa, where healthcare systems are already strained due to high demand and limited resources.
• The article calls for further research into the relationship between substance use and acute psychiatric episodes, especially in the South African and broader African context.

Alt-text: Unlabelled box

## Background

Mental illness and substance abuse are global public health crises, each independently contributing to high rates of morbidity and mortality [1,2]. Their co-occurrence worsens clinical outcomes through medication nonadherence, reduced healthcare access, and the neuropsychiatric effects of substances on preexisting mental health conditions [3]. These intertwined challenges strain healthcare systems, particularly in low- and middle-income countries (LMICs), where over 80 % of individuals with mental disorders reside [4,5]. Mental illness and substance use account for 8.8 % and 16.6 % of the disease burden in LMICs, respectively [1,4].

Patients with psychiatric conditions require substantial hospital and community resources, contributing to financial costs, Emergency Centre (EC) overcrowding, prolonged inpatient boarding times, and psychological stress on caregivers and providers [6,7]. Notably, many patients initially diagnosed with acute psychosis in ECs may instead have substance-induced psychiatric episodes or worsened preexisting disorders [8].

Substance use frequently co-occurs with mental health disorders [9,10]. In the USA (2021), 21.9 % of individuals aged ≥12 reported illicit drug use, while 7.6 % had concurrent mental health and substance use disorders [11]. Adolescents who experiment with substances later in adolescence face fewer long-term risks than early users [10,12]. Conversely, the number of older adults with substance use disorders, predominantly involving alcohol, is rising [11,13]. International trial data suggests a gender disparity in the presentation of psychosis, with males exhibiting higher rates of substance use [14], earlier onset of symptoms, and poorer functional outcomes [15,16]. Males who abuse substances likely contribute to elevated hospital readmission rates [16].

The repeated incidents of mental health crises among patients put a strain on available resources [[17], [18], [19]], yet the role of substances in repeat hospitalizations remains understudied [8]. In South Africa, psychiatric units report a high substance use disorder rate [20,21], but district-level hospital data are limited [22]. At Victoria Hospital, over 50 % of acute psychiatric referrals test positive for psychoactive substances [23].

This study addresses the lack of local data on mental health and substance use comorbidity in a South African district hospital by investigating the measured burden of substance use among psychiatric inpatients at Victoria Hospital; the proportion of psychiatric referrals testing positive for psychoactive substances; and age- and sex-related trends in substance use among positive cases.

## Methods

### Study design

This study was a single-centre, retrospective, descriptive analysis.

### Study setting

The study was conducted at Victoria Hospital, a 203-bed public sector district hospital located in the southwestern metropole of Cape Town, South Africa. The hospital serves a population of approximately 620,000 people [24], predominantly residing in the southern suburbs of Cape Town. Most of the population served are from low- to middle-income households. The EC provides comprehensive medical care to 2500 to 3000 patients each month, averaging 85 to 100 patients per day, based on data from the Hospital and Emergency Centre Tracking Information System (HECTIS) [25]. Victoria Hospital has 34 dedicated inpatient beds for psychiatric care. It acts as a referral facility for 10 government institutions, including primary healthcare clinics and day hospitals, as well as private facilities within the drainage area. Patients are referred for inpatient psychiatric assessment and a 72-hour observation under the Mental Healthcare Act if applicable.

### Study population and sampling strategy

All patients who presented to the EC at Victoria Hospital over the six-month study period (October 2022 to March 2023) and who were referred to the inpatient psychiatry service after their initial consultation in the EC were included. A convenience sample of 500 – 600 patients was used based on average monthly psychiatry referrals extracted from HECTIS. Minors younger than 12 years and those referred for nonfatal suicidal behaviour (e.g.: intentional overdose attempt) were excluded from the study. Patients referred with nonfatal suicidal behaviour were excluded as the interpretation of results can be challenging without a detailed review of each specific case and the substances involved.

All patients visiting the EC are captured in an online electronic patient information system known as HECTIS. This system, implemented by the Western Cape Department of Health, tracks patients throughout their stay in the EC. Each patient assessed by a doctor is assigned an ICD-10 code (indicating the diagnosis) and a disposition plan (e.g., discharged home or referred to an inpatient service), both of which are recorded in HECTIS.

According to current psychiatric departmental procedures at Victoria Hospital, urine samples are collected from all patients referred to psychiatry. Urine drug testing aids in planning the management and disposition of these patients. Victoria Hospital relies on the pharmacology laboratory at Groote Schuur Hospital, which acts as a tertiary-level referral laboratory for district hospitals, for urine psychoactive drug screening. At this laboratory, a standard 5-drug urine toxicology panel is performed using a lateral flow assay technique, which includes methamphetamine, cannabis, opioids, benzodiazepines, and cocaine.

Psychoactive substances not included in the standard five-drug urine toxicology panel are tested using liquid chromatography/mass spectrometry (LC/MS). LC/MS is not routinely performed for patients admitted with psychiatric diagnoses due to the high costs of these tests; it is primarily reserved for forensic cases or conducted upon physician request. Occasionally, the Victoria Hospital EC receives referred forensic patients with acute mental disorders which may necessitate LC/MS analysis to detect additional psychoactive substances in these cases. However, during our study period, no patients underwent LC/MS testing.

### Data collection

The principal investigator collected the data, which was recorded in an Excel spreadsheet and subsequently checked for errors and omissions. The data was managed and stored in a password-protected cloud-based registry.

Data collection was conducted in three steps:

Step One: A data extraction tool was developed for this study and included the following variables: Hospital number; Sex (male/female/unspecified); Age; Diagnosis (primary and any secondary ICD-10 code (s)); Drug test result: positive/negative; If positive, which drug (s) tested positive (the “Other” category indicates LC/MS analysis, if available); Repeat visit (yes/no).
Step Two: A search of the HECTIS database was performed to identify all patients referred to Psychiatry. A database was created using the variables specified in the data extraction tool.
Step Three: A search using the online results portal of Groote Schuur Hospital's pharmacology laboratory was conducted. Patient hospital numbers were used to extract the following variables: urine drug test results (positive/negative) and, if positive, the specific substance (s) that tested positive. This data was captured in the electronic database.

To ensure internal validity of the collected data, the principal investigator cross-checked a small sample by randomly selecting 60 (10 %) hospital numbers from the included patients to determine if the data were correct. No errors were detected.

### Analysis

Non-parametric numeric variables were summarised using the median and interquartile range, while categorical variables were summarised using frequencies and proportions. To compare numeric variables across different strata, the Mann–Whitney U test was used. For categorical variables, the Chi-squared test was employed, applying Yates’ continuity correction for small frequencies. Unless stated otherwise, all statistical tests were two-sided, with statistical significance defined as *p* < 0.05. Data analysis was conducted using R Statistical Software (version 4.3.0) [26].

## Ethics approval

Ethics approval was obtained from the Human Research Ethics Committee of the University of Cape Town (Ref. No. 201/2024), the Western Cape Department of Health and Wellness (Ref. No. WC_202,405_024), and the management and Ethics Committee of Victoria Hospital. Written permission to access and extract data using Groote Schuur Hospital's Pharmacology Laboratory's online results portal was obtained from the current University of Cape Town Head of Division: Clinical Pharmacology. This study was considered low risk because data was anonymized at the time of collection, it was analyzed without specific identifiers, there was no direct patient contact or intervention, and patient care was not affected by the study.

## Results

**Fig. 1:**
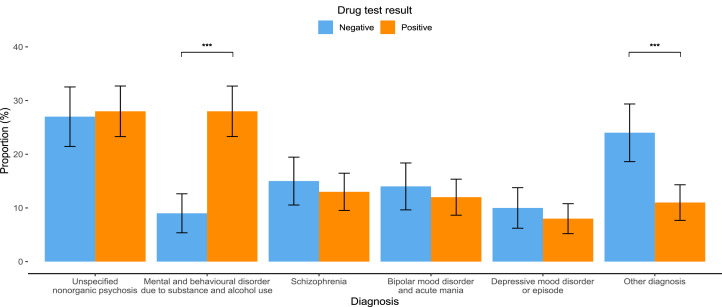
Diagnosis by test result.

**Fig. 2:**
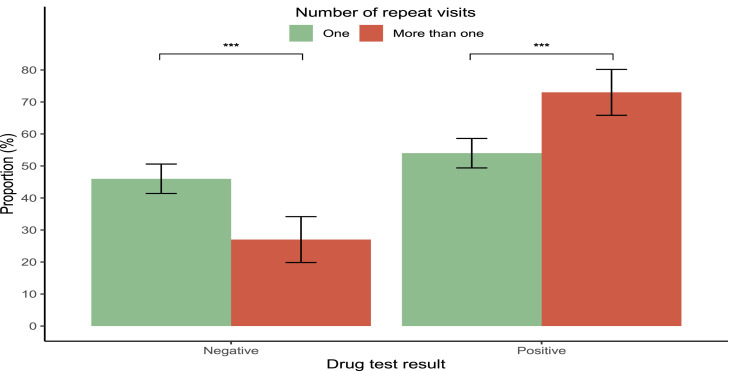
Drug test result by number of visits.

Fifty-nine percent of patients referred to psychiatric inpatient services tested positive on urine drug screening (Table 1). On average, patients who tested positive were younger, with an average age of 33 years, compared to 36 years for those who tested negative (*p* = 0.005; Suppl Fig. 1). Patients with positive drug tests were more likely to be diagnosed with mental and behavioural disorders related to substance and alcohol use (28 %) compared to those with negative tests (9 %) (*p* < 0.001). In contrast, diagnoses of other disorders were more prevalent among patients with negative drug tests (24 %) compared to those with positive tests (11 %) (*p* < 0.001; Fig. 1). Additionally, a greater proportion of patients with a positive drug test had multiple visits, with 30 % of patients returning to the hospital more than once during the study period, compared to 16 % of those who tested negative (*p* < 0.001; Fig. 2).

**Table 1:** Summary of inpatient psychiatry visits overall and stratified according to sex, drug test results and number of visits. Numeric^1^ variables are summarised as the median with the interquartile range (IQR) in brackets. Categorical ^2^ variables are summarised as frequencies with proportions in brackets.

Characteristic	Overall (*N* = 597)	Female (*N* = 248)	Male (*N* = 349)	p-value	Drug test result: Overall (*N* = 597)	Drug test result: Negative (*N* = 246)	Drug test result: Positive (*N* = 351)	p-value	Number of visits: Overall: (*N* = 597)	Number of visits: One (*N* = 451)	Number of visits: More than one (*N* = 146)	P-value
**Age (years)**	34 (27, 44)	36 (28, 47)	33 (27,42)	0.047	34 (27, 44)	36 (28, 50)	33 (27, 42)	0.005	34 (27, 44)	35 (27, 47)	31 (27, 38)	0.002
**Sex**								0.002				0.678
Female					248 (42 %)	121 (49 %)	127 (36 %)		248 (42 %)	190 (42 %)	58 (40 %)	
Male					349 (58 %)	125 (51 %)	224 (64 %)		349 (58 %)	261 (58 %)	88 (60 %)	
**Diagnosis**												
Unspecified nonorganic psychosis	166 (28 %)	53 (21 %)	113 (32 %)	0.004	166 (28 %)	66 (27 %)	100 (28 %)	0.724	166 (28 %)	129 (29 %)	37 (25 %)	0.511
Mental and behavioral disorder due to substance and alcohol use	122 (20 %)	41 (17 %)	81 (23 %)	0.059	122 (20 %)	23 (9 %)	99 (28 %)	<0.001	122 (20 %)	79 (18 %)	43 (29 %)	0.003
Schizophrenia	81 (14 %)	20 (8 %)	61 (17 %)	0.001	81 (14 %)	37 (15 %)	44 (13 %)	0.448	81 (14 %)	58 (13 %)	23 (16 %)	0.454
Bipolar mood disorder and acute mania	76 (13 %)	58 (23 %)	18 (5 %)	<0.001	76 (13 %)	35 (14 %)	41 (12 %)	0.427	76 (13 %)	56 (12 %)	20 (14 %)	0.794
Depressive mood disorder or episode	52 (9 %)	26 (10 %)	26 (7 %)	0.251	52 (9 %)	25 (10 %)	27 (8 %)	0.365	52 (9 %)	47 (10 %)	5 (3 %)	0.015
Other diagnosis	100 (17 %)	50 (20 %)	50 (14 %)	0.077	100 (17 %)	60 (24 %)	40 (11 %)	<0.001	100 (17 %)	82 (18 %)	18 (12 %)	0.129
**Drug test result**				0.002								<0.001
Negative	246 (41 %)	121 (49 %)	125 (36 %)						246 (41 %)	207 (46 %)	39 (27 %)	
Positive	351 (59 %)	127 (51 %)	224 (64 %)						351 (59 %)	244 (54 %)	107 (73 %)	
Tested positive for Benzodiazepines	92 (26 %)	46 (36 %)	46 (21 %)	0.002					92 (26 %)	66 (27 %)	26 (24 %)	0.684
Tested positive for Cannabis	210 (60 %)	56 (44 %)	154 (69 %)	<0.001					210 (60 %)	148 (61 %)	62 (58 %)	0.720
Tested positive for Methamphetamine	166 (47 %)	55 (43 %)	111 (50 %)	0.310					166 (47 %)	104 (43 %)	62 (58 %)	0.011
Tested positive for Opioids	25 (7 %)	12 (9 %)	13 (6 %)	0.289					25 (7 %)	24 (10 %)	1 (1 %)	0.006
Tested positive for Cocaine	4 (1 %)	0	4 (2 %)	0.301					4 (1 %)	2 (1 %)	2 (2 %)	0.588
**Number of different substances in positive test**				0.103								0.695
1	229 (65 %)	92 (72 %)	137 (61 %)						229 (65 %)	159 (65 %)	70 (65 %)	
2	98 (28 %)	28 (22 %)	70 (31 %)						98 (28 %)	70 (29 %)	28 (26 %)	
3	24 (7 %)	7 (6 %)	17 (8 %)						24 (7 %)	15 (6 %)	9 (8 %)	
**Number of repeat visits**				0.678				<0.001				
Once	451 (76 %)	190 (77 %)	261 (75 %)		451 (76 %)	207 (84 %)	244 (70 %)					
More than once	146 (24 %)	58 (23 %)	88 (25 %)		146 (24 %)	39 (16 %)	107 (30 %)

Error bars: 95 % CI, * *P* ≤ 0.05 ** *P* ≤ 0.01 *** *P* ≤ 0.001

Error bars: 95 % CI, * *P* ≤ 0.05 ** *P* ≤ 0.01 *** *P* ≤ 0.001

Male patients accounted for a higher number of psychiatry visits compared to female patients, with 58 % being male and 42 % female (Table 1). On average, male patients were younger than female patients, with ages of 33 years and 36 years, respectively (*p* = 0.047; Suppl Fig. 1). The diagnosis of unspecified nonorganic psychosis was more common in males (32 %) than in females (21 %) (*p* = 0.004). Similarly, schizophrenia was more prevalent in males, affecting 17 % compared to 8 % of females (*p* = 0.001). In contrast, bipolar mood disorder and acute mania were more frequently diagnosed in females compared to males, with rates of 23 % and 5 %, respectively (*p* < 0.001; Suppl Fig. 2).

**Fig. 3:**
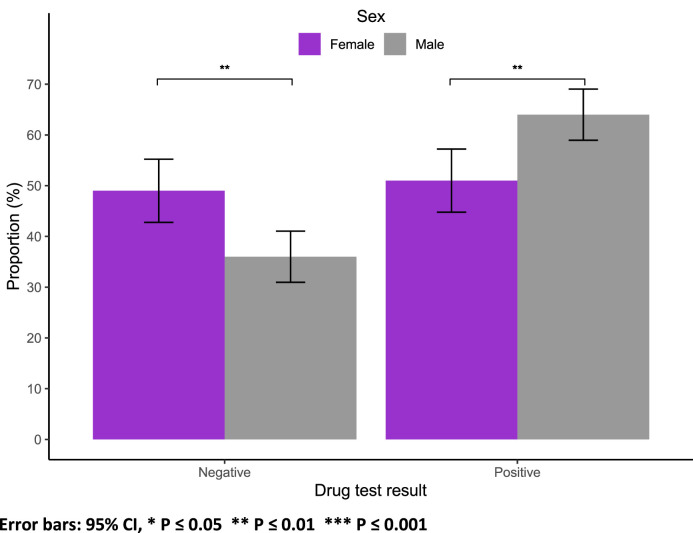
Drug test result by sex.

In terms of substance use, a greater proportion of males (64 %) tested positive compared to females (51 %) (*p* = 0.002; Fig. 3). Among those who tested positive for substances, cannabis use was more prevalent among males (69 % vs. 44 %, *p* < 0.001), while a larger percentage of females used benzodiazepines (36 % vs. 21 %, *p* = 0.002; Suppl Fig. 3).

Error bars: 95 % CI, * *P* ≤ 0.05 ** *P* ≤ 0.01 *** *P* ≤ 0.001

Similar proportions of males and females had repeat visits, with 25 % of males and 23 % of females demonstrating this pattern (*p* = 0.678). No sex-specific differences were found regarding diagnoses related to mental and behavioural disorders due to substance and alcohol use (*p* = 0.059), depressive mood disorders or episodes (*p* = 0.251), or other diagnoses (*p* = 0.077). For patients who tested positive for substances, the prevalence of methamphetamine, opioids, and cocaine was not statistically significantly different between males and females (*p* > 0.05). Additionally, while a higher percentage of males (61 %) tested positive for one substance compared to females (72 %), this difference was not statistically significant (*p* = 0.103).

**Fig. 4:**
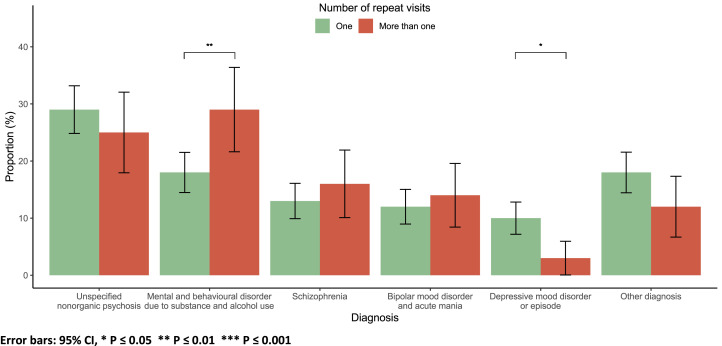
Diagnosis by number of visits.

Most patients (76 %) had one inpatient psychiatry visit (Table 1). On average, patients with a single visit were older, at 35 years, compared to those with more than one visit, who averaged 31 years (*p* = 0.002; Suppl Fig. 1). A diagnosis of mental and behavioural disorders related to substance and alcohol use was linked to repeat visits (*p* = 0.003). In contrast, a diagnosis of depressive mood disorder or episode was associated with one visit (*p* = 0.015; Fig. 4).

Error bars: 95 % CI, * *P* ≤ 0.05 ** *P* ≤ 0.01 *** *P* ≤ 0.001

**Fig. 5:**
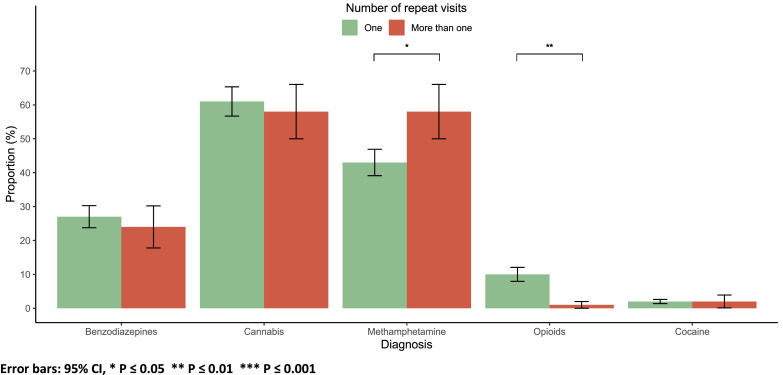
Substance by number of visits.

Methamphetamine use was more prevalent among patients with repeat visits (58 % vs. 42 %, *p* = 0.011), while opioid use was more common among those with only one visit (10 % vs. 1 %, *p* = 0.006). There were no significant differences in the use of benzodiazepines, cannabis, cocaine, or the presence of multiple substances detected based on the number of visits (*p* > 0.05; Fig. 5).

Error bars: 95 % CI, * *P* ≤ 0.05 ** *P* ≤ 0.01 *** *P* ≤ 0.001

## Discussion

This study offers a comprehensive overview of the prevalence and patterns of psychoactive substance use among psychiatric in-patients at Victoria Hospital in Cape Town. Our findings provide significant insights into the demographic characteristics of the patient population, the burden of substance use, and the relationships between substance use and psychiatric diagnoses.

### Prevalence of substance use

Fifty-nine percent of psychiatry inpatients tested positive for psychoactive substances, highlighting the substantial burden of substance use in this demographic. This high prevalence aligns with existing literature [1,27] that indicates a strong association between psychiatric disorders and substance use, particularly in emergency settings [4,5,9,11,27]. This finding emphasises the urgent need for integrated treatment approaches that address both psychiatric and substance use disorder [28], especially given the high rates of comorbidity observed.

### Demographic trends

Our findings suggest a higher prevalence of substance use among male patients (64 %) compared to females (51 %). This supports previous studies indicating a gender disparity in substance use within psychiatric populations [14,15]. Notably, males not only exhibited a higher rate of substance use but also a greater prevalence of diagnoses such as schizophrenia and unspecified nonorganic psychosis. In contrast, females were more frequently diagnosed with bipolar mood disorder and acute mania. This may reflect underlying social and biological factors influencing the manifestation of these disorders and substance use behaviours [14,15]

Age-related trends also emerged, with younger patients (average age 33) more likely to test positive for substances than older patients (average age 36). This aligns with research suggesting that younger individuals may engage in riskier behaviours, including substance use, and may be more prone to presenting to emergency services during psychiatric crises [16]

### Substance-specific findings

Cannabis was the most commonly detected substance, with 60 % of patients testing positive. Methamphetamine were found in 47 % of the patients, while approximately 26 % tested positive for benzodiazepines. A smaller percentage, 7 %, showed positive results for opioids, and cocaine use was minimal, with only 1 % of patients testing positive. This pattern of substance use is likely linked to socioeconomic circumstances within our patient population. Cannabis and methamphetamine, likely being cheaper and easier to obtain, are more commonly used. This pattern reflects broader trends of substance use in the Western Cape [29,30].

Among those who tested positive for substances, 65 % had only one substance detected, 28 % had two, and 7 % had three substances. Cannabis was the most prevalent substance among males, whereas benzodiazepines were more frequently used by females. This gender-specific difference in substance use may suggest varying coping mechanisms or social contexts that could influence these behaviours [31]. These patterns highlight the importance of addressing gender-specific substance use issues within psychiatric settings, as they can significantly impact treatment outcomes.

It is important to note that the Cannabis for Private Purposes Act, which decriminalises the private use of cannabis for adults in South Africa, was signed into law on 28 May 2024 [32]. While our study was conducted before this legislation was passed, the prevalence of cannabis use among young adults may increase as private consumption becomes more socially accepted.

### Patterns of repeated visits

The higher proportion of repeat visits among patients testing positive for substances (30 %) as compared to those with negative tests (16 %) indicates a concerning cycle of acute psychiatric crises linked to substance use. This underscores the need for ongoing care and support mechanisms to help break this cycle [17], especially for younger individuals who are more frequently re-admitted. The relationship between diagnoses and visits is also noteworthy: patients diagnosed with mental and behavioural disorders due to substance use were more likely to have multiple visits, indicating a need for more intensive and sustained intervention strategies.

## Limitations

The retrospective nature of the study only allows for the description of existing data and relies heavily on accurate data capturing. Patients might have been missed during the study period if they were incorrectly diagnosed or processed in the HECTIS system. However, clinicians are experienced in using HECTIS, which should mitigate this limitation. Furthermore, patients presenting to the EC with substance-induced psychiatric symptoms who do not require psychiatric admission have been excluded, though we believe this to have minimal impact on our study findings.

It has been noted that there is some cross-reactivity between certain substances and medications in urine toxicology analysis, resulting in a possibility of false-positive results [33,34]. As the nature of the study did not include chart reviews, we could not determine whether the patients that tested positive for benzodiazepines were sedated with benzodiazepines before urine sample collection. Some EMS personnel are authorized to administer benzodiazepines in the prehospital setting. It's important to consider that patients referred from other centres may have already received these medications prior to transfer. This means that observed positive benzodiazepine results could be due to medical intervention rather than recreational use, which limits the significance of this finding.

Drugs of abuse have a limited detection window in urine, blood, and saliva. In urine, this detection time typically ranges from 1.5 to 4 h, though it can be longer in chronic users [35]. Therefore, if a patient has used drugs more than a few hours before being tested, they may receive a negative result.

We recognise that the standard five-drug urine toxicology panel detects the most commonly abused psychoactive substances. However, other psychoactive substances may go undetected without additional testing. We believe that the routine use of these undetected substances is minimal in our population and is therefore unlikely to significantly impact the results. Additionally, we trust that the admitting team would request further testing for psychoactive substances if clinically appropriate, and those results would be documented.

We also acknowledge that the traditional male-female classification system, which does not account for other gender identities, may limit the applicability of our findings to broader population groups.

Future research should consider longitudinal studies to track substance use and psychiatric outcomes more effectively, thereby adopting a systems approach to address this complex issue. Additionally, exploring the socio-economic, geospatial, and cultural factors influencing substance use in this population could yield a deeper understanding of the underlying issues and help tailor interventions to meet the specific needs of the community.

## Conclusion

This study underscores the significant burden of psychoactive substance use among psychiatric inpatients in Cape Town. The identified demographic and substance-specific trends provide critical information for healthcare providers, highlighting the need for integrated treatment strategies that address both psychiatric and substance use disorders. Ongoing research and targeted interventions will be essential to improving outcomes for this vulnerable population.

## Dissemination of results

The results of this study will be shared with the clinical and management teams of Victoria Hospital and with the staff involved in the study. This will be done via an informal presentation.

## Declaration of competing interest

The authors declare that they have no known competing financial interests or personal relationships that could have appeared to influence the work reported in this paper.

## Supplementary materials

Image, application 1
