# Supplementary material for: Psychoactive substance use among psychiatric in-patients presenting to the Emergency Centre of a district hospital in Cape Town, South Africa. A retrospective descriptive study
Source: Afr J Emerg Med. 2025 Mar 21;15(2):577–83. doi: 10.1016/j.afjem.2025.02.006 (PMC11982960; doi:10.1016/j.afjem.2025.02.006)
Supplement: Supplementary file 1 [file mmc1.pdf]

Electronic Addendum

Suppl Fig. 1 - **Histogram of age**

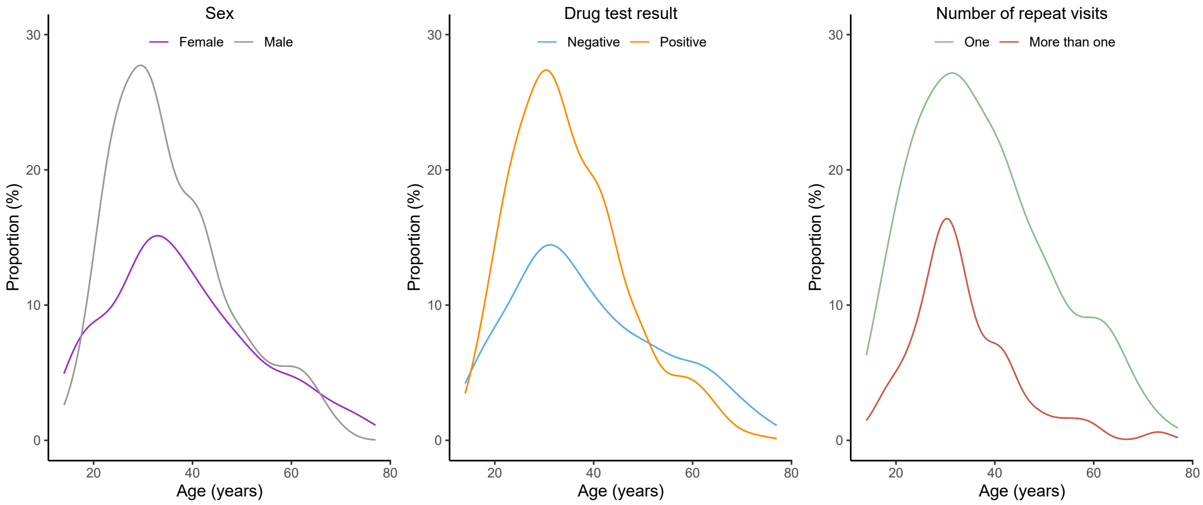

Suppl Fig. 2 - **Diagnosis by sex**

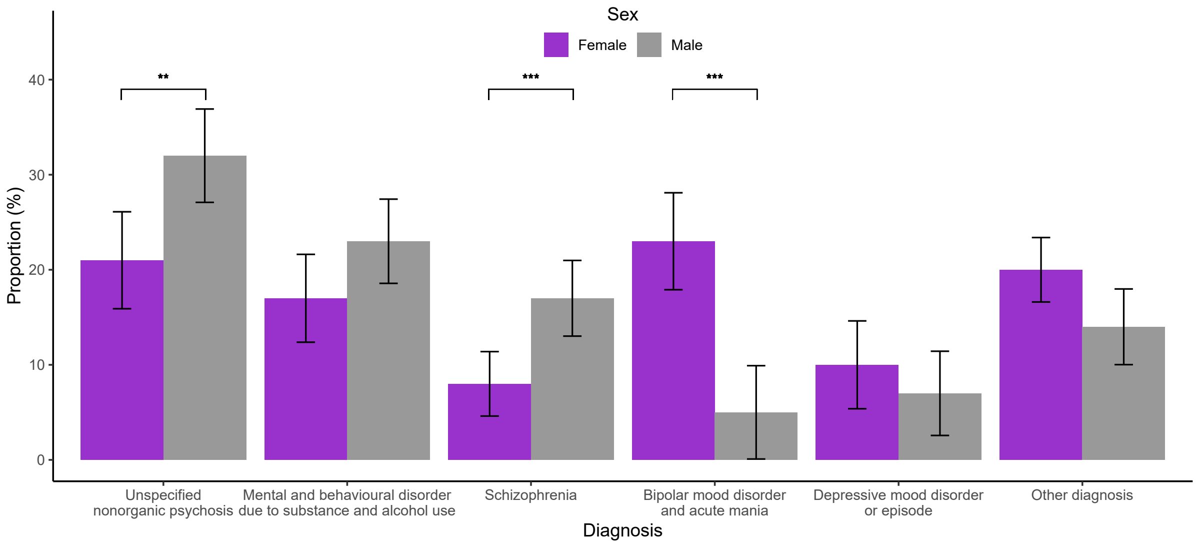

Error bars: 95% CI, * P ≤ 0.05 ** P ≤ 0.01 *** P ≤ 0.001

Suppl Fig. 3 - **Substance by sex**

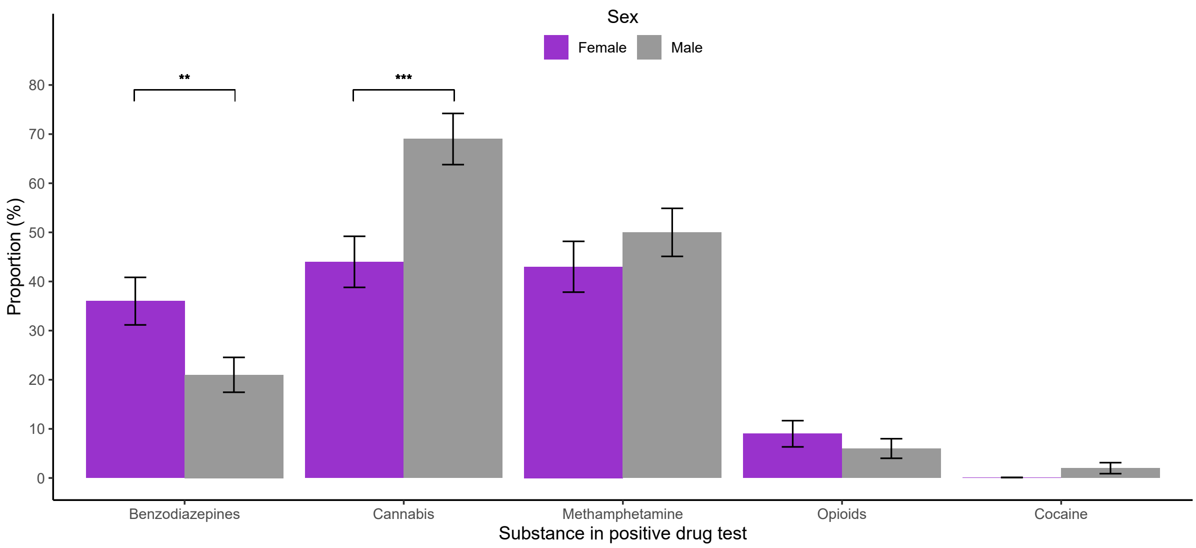

Error bars: 95% CI, * P ≤ 0.05 ** P ≤ 0.01 *** P ≤ 0.001
